# Acute Gastric Necrosis Secondary to Mesenteric Ischemia: A Case Report and Literature Review

**DOI:** 10.7759/cureus.76680

**Published:** 2024-12-31

**Authors:** Julio González García, Belen Domingo Cruz Hernandez, Luis Fernando Pérez Solis, Natalia A Pucheta Hernández, Jesus Eduardo Trujillo Rodríguez, Victor M Martinez Bravo

**Affiliations:** 1 Surgery, Instituto Mexicano del Seguro Social, Colima, MEX; 2 Surgery, Universidad Xochicalco Ensenada, Ensenada, MEX; 3 Medicine, Universidad Nacional Autónoma de México, Mexico City, MEX; 4 Nutrition, Hospital Regional de Alta Especialidad Instituto de Seguridad y Servicios Sociales de los Trabajadores del Estado B Veracruz, Veracruz, MEX; 5 Surgery, Universidad Cristóbal Colón, Veracruz, MEX; 6 General Surgery, Hospital Regional de Alta Especialidad Instituto de Seguridad y Servicios Sociales de los Trabajadores del Estado B Veracruz, Veracruz, MEX

**Keywords:** eating disorders, fundoplication, gastric dilation, gastric necrosis, intestinal obstruction

## Abstract

Acute gastric dilation and necrosis, although rare, are most commonly associated with eating disorders. We present a case of a patient with a history of prior fundoplication and complete intestinal obstruction, which led to severe gastric dilation and subsequent gastric necrosis. The condition was successfully managed through partial gastrectomy. Initial management involved gastric decompression with a nasogastric tube and fluid resuscitation. Surgical intervention remains the definitive treatment in most reported cases. Early diagnosis and timely intervention are critical to improving outcomes and minimizing morbidity and mortality.

## Introduction

Acute gastric dilation (AGD) is a rare but potentially life-threatening condition that can result from various etiologies, including eating disorders, pyloric spasm, gastric volvulus, gastroparesis, and gastrointestinal tumors. However, gastric dilation caused by acute intestinal obstruction is infrequent, and progression to massive gastric dilation with ischemia and necrosis is an exceptionally rare occurrence, associated with high mortality rates [1,2].

The phenomenon of acute gastric dilation was first described in 1833. Among its complications, gastric perforation and necrosis represent the most severe outcomes [3]. Clinically, patients with gastric ischemia typically present with a history of symptoms indicative of gastric distension. Key manifestations include upper gastrointestinal bleeding, ileus, massive abdominal distension, inability to vomit (in cases of fundoplication), signs of peritonitis, acute abdominal pain, and nausea [4-6].

Diagnostic evaluation begins with a supine abdominal X-ray, which often reveals significant gastric distension or aerial meniscus in case of perforation. Computed tomography (CT) scans of the abdomen can further delineate the underlying etiology and detect signs of gastric necrosis. These include diffuse or focal thickening of the gastric wall, often with hypoattenuation suggesting edema or ischemia, and the absence of mucosal enhancement, indicating compromised blood flow. Pneumatosis gastrica, the presence of gas within the gastric wall, is a hallmark feature, and portal venous gas may also be observed, resulting from bacterial translocation and transmural necrosis. Severe gastric dilation with fluid or gas accumulation is often present, accompanied by perigastric fat stranding, indicative of inflammation. In advanced cases, free intraperitoneal air may signal gastric perforation. Additionally, the loss of normal differentiation between the gastric wall layers due to ischemia further supports the diagnosis. These findings, when correlated with clinical symptoms, are crucial for prompt diagnosis and management [6].

Initial management involves gastric decompression using a nasogastric tube and fluid resuscitation. Definitive treatment in most cases is surgical, with partial or total gastrectomy being the most effective intervention. Delayed surgical treatment significantly increases mortality, with rates reported as high as 50-80% [1-12]. Despite an extensive literature review, no prior case reports have been identified linking intestinal obstruction to gastric necrosis. This report describes a unique case of gastric necrosis secondary to acute gastric dilation (AGD) caused by intestinal obstruction due to an internal hernia in a patient with a history of prior fundoplication.

Gastric necrosis resulting from dilation is an exceedingly rare condition, with limited cases documented in the literature [1-6]. Although it is most commonly associated with eating disorders, timely recognition of this condition and its complications is critical for reducing its high mortality rate. Awareness of the clinical presentation and appropriate management can significantly improve patient outcomes.

## Case presentation

A 56-year-old female with a history of arterial hypertension, acid-peptic disease, and hypothyroidism, and a surgical history of Nissen fundoplication performed 10 years ago for gastroesophageal reflux disease (GERD) with esophagitis and a hiatal hernia, presented to the emergency department with a 48-hour history of symptoms. She reported sudden-onset epigastric abdominal pain, graded as 8/10 on the verbal analog scale, accompanied by nausea.

Examination findings

The patient's vital signs on examination were as follows: blood pressure was 122/83 mmHg, heart rate was 119 beats per minute, respiratory rate was 20 breaths per minute, and temperature was 36.7°C. Neurological and cardiorespiratory examinations revealed no abnormalities. On abdominal examination, there was significant distension, tenderness on both superficial and deep palpation, positive rebound tenderness, and clear evidence of peritoneal irritation.

Laboratory and imaging findings

**Figure 1:**
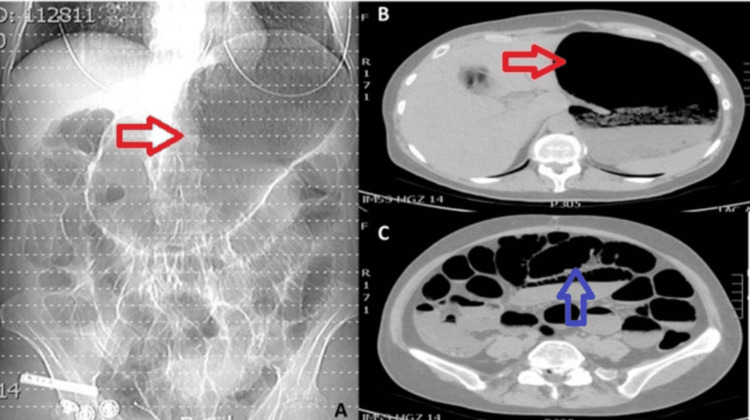
Contrast-enhanced CT of the abdomen and pelvis. (A) Abdominal X-ray showing gastric dilation and dilated bowel loops (red arrows). (B) Simple tomography of the abdomen axial section showing gastric dilation (red arrow) and (C) dilation of intestinal loops (blue arrow).

Paraclinical tests revealed the following: hemoglobin level of 18 g/dL, platelets at 198,000/mm³, leukocytes at 9.4 x 10³/µL, neutrophilia at 80.7%, creatinine at 1.3 mg/dL, an elevated C-reactive protein (CRP) of 14 mg/L (normal: <5 mg/L), and arterial blood gas analysis showing elevated lactate levels of 4.5 mmol/L (normal: <2 mmol/L). Radiography indicated significant gastric and intestinal loop distension with multiple air-fluid levels. A contrast-enhanced CT of the abdomen and pelvis (CTAP) revealed marked gastric dilation, distension of small bowel loops with multiple hydro-aerial levels, apparent gastric and intestinal pneumatosis, and findings suggestive of ischemia/necrosis (Figures 1A-1C). The general surgery team was consulted for suspected intestinal obstruction. Based on the clinical presentation and imaging findings, a diagnosis of acute abdomen, likely secondary to intestinal obstruction, was made. Emergency surgery was promptly undertaken.

**Figure 2:**
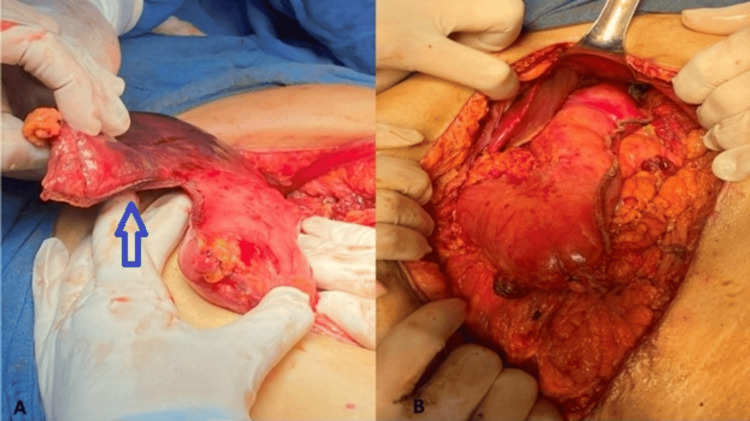
Exploratory laparotomy and ischemic partial segment resection. (A) Exploratory laparotomy showing necrosis of the greater curvature of the stomach. (B) Ischemic partial segment resection of the stomach in the sleeve.

An exploratory laparotomy was performed, revealing approximately 1.5 L of inflammatory fluid in the abdominal cavity. The source of the obstruction was identified as an internal hernia located 30 cm proximal to the ileocecal valve, causing ischemia in a 10 cm segment of the distal ileum. The affected segment showed evidence of ischemia but recovered normal coloration and peristalsis during the procedure, negating the need for bowel resection. In the stomach, necrosis was observed in 60% of its structure, involving the greater curvature, fundus, and both anterior and posterior aspects (Figures 2A, 2B). The site of the previous Nissen fundoplication was evaluated and found to be intact, with no evidence of dehiscence or dysfunction.

**Figure 3:**
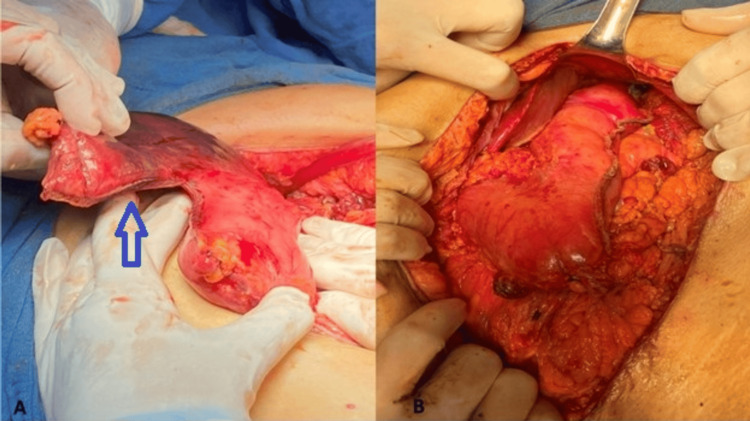
Resection and partial surgical images. (A) Initiation of the staple line at the time of resecting ischemic tissue (blue arrow). (B) Stomach after partial surgical resection.

Given the extent of necrosis, a vertical partial gastrectomy was performed. This was achieved using a 65 mm linear stapler with a blue cartridge, ensuring precise resection of the ischemic gastric segment. The abdominal cavity was thoroughly irrigated to remove residual inflammatory fluid and prevent further contamination. Two drains were placed - one in the left subphrenic space and the other in the pelvic cavity - for postoperative monitoring and drainage (Figures 3A, 3B).

The patient was transferred unstable to an intermediate therapy area with ventilatory support and vasoactive amines norepinephrine, later vasopressor withdrawal was achieved in the first hours after surgery and mechanical ventilation was disengaged on the fourth postoperative day. During hospitalization, evaluation by hematology and cardiology was requested for suspected cardiovascular etiology, coagulopathies, and immunorheumatologic causes, with the following profiles: protein S 58%, protein C 40%, IgM anticardiolipin <2 U/mL, IgG anticardiolipin <2 U/mL, antiphospholipid antibodies <1 U/mL, lupus anticoagulant 1.66 U/mL, IgG antibeta-2 glycoprotein <2 U/mL, IgM antibeta-2 glycoprotein <1 U/mL, alpha-fetoprotein 3 ng/mL, carcinoembryonic antigen 1.73 ng/mL, CA 19-9 9.42 U/mL, CA 125 55 U/mL, complement C4 25 mg/dL, antinuclear antibodies (ACAs) 1:80, anti-Smith ACAs <2 U/mL (measured to rule out a neoplastic process as a secondary cause of the ischemia).

**Figure 4:**
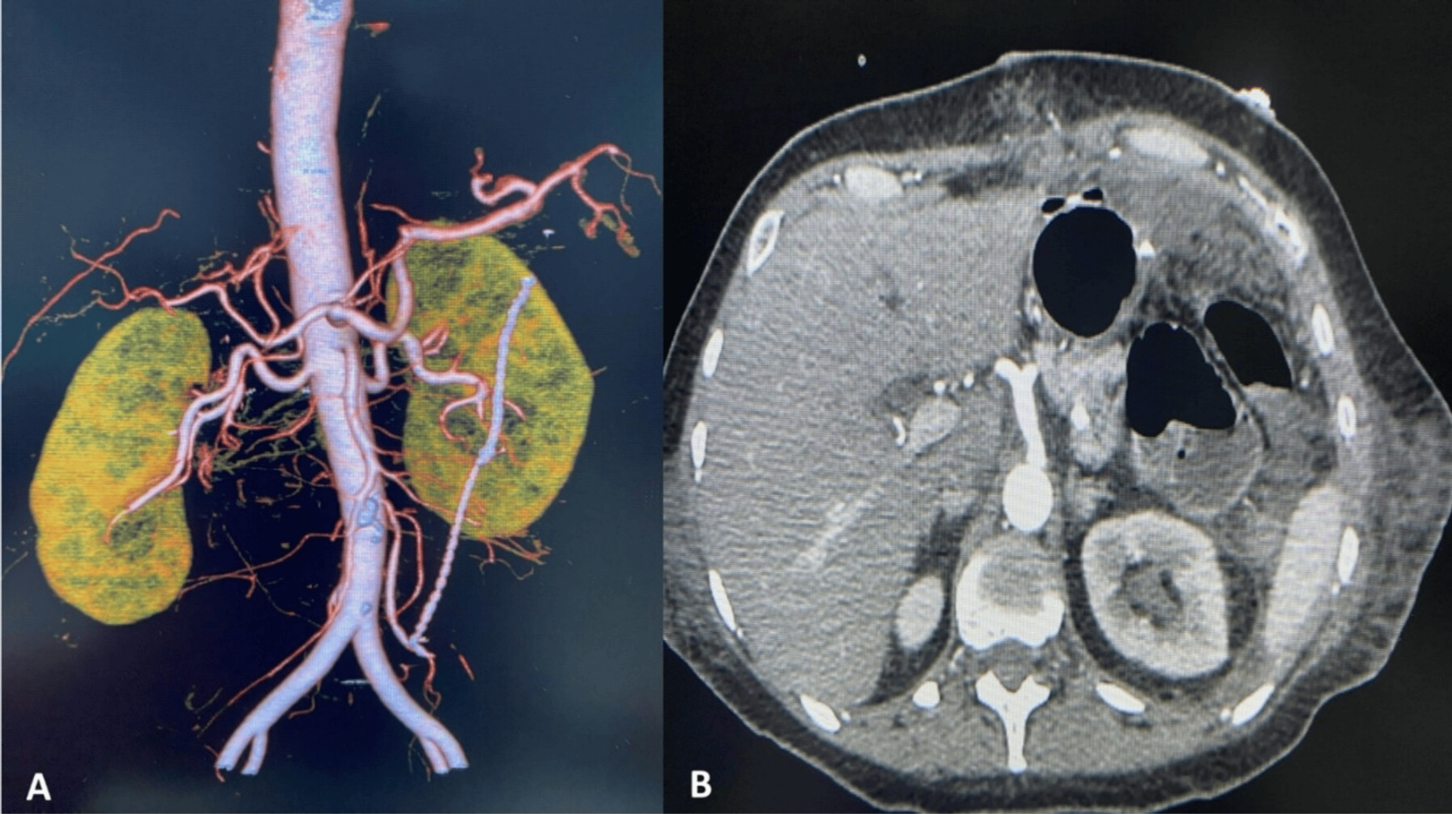
Axial and sagittal CT images of acute gastric necrosis with clear demarcation. (A) Abdominal CT angiography does not show vascular alterations in the abdominal trunks. (B) Axial tomography angiography shows integrity of the celiac trunk.

A result of D-dimer with a value of 3919 ng/mL (normal range: <500 ng/mL), fibrinogen 821 U/L (normal range: 20-60 U/L) was reported, for which thromboembolism was suspected and pulmonary and abdominal angiography was performed, which ruled out pulmonary thrombosis. The abdominal aortic trunk and left gastric artery show adequate opacification and proper trajectory (Figures 4A, 4B).

**Figure 5:**
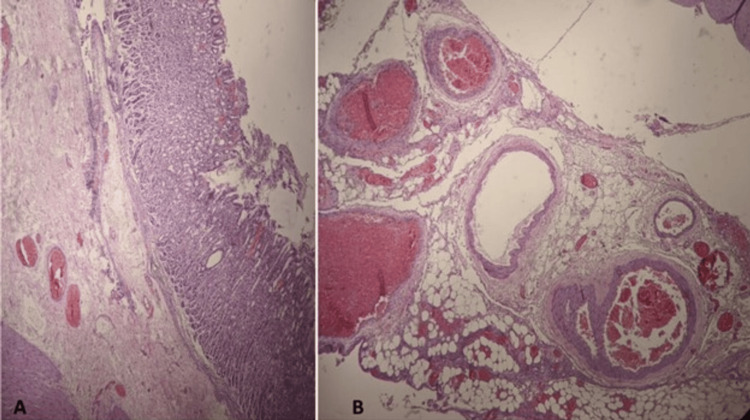
Intraoperative images displaying necrotic gastric tissue and postresection anatomy. (A, B) Panmural segmental necrosis in 20% of the tissue, diffuse ischemic changes, acute vasculopathy. No evidence of microorganisms or malignancy.

Histopathological examination reported segmental panmural necrosis in 20% of the tissue, diffuse ischemic changes, and acute vascular disease. No evidence of microorganisms or malignancy was seen (Figures 5A, 5B). A stable patient was discharged on day 13 postoperatively.

## Discussion

Acute gastric dilation can be secondary to multiple causes, either due to accumulation of food, fluid or air, pyloric spasm, gastric volvulus, gastroparesis, gastrointestinal tumors, and eating disorders, the latter cause in patients with bulimia and anorexia, it is a frequent condition in multiple case reports [1,2].

It should be noted that gastric dilation secondary to acute intestinal obstruction as a cause of gastric ischemia is infrequent, and this sequence of clinical events is a rare condition with high mortality. Although an exhaustive search was conducted in the database, few reports of cases associating intestinal obstruction with gastric necrosis were identified. However, there are case reports of gastric necrosis in patients who are unable to burp or vomit due to Nissen-type fundoplication, a condition present in this patient. These case reports, however, mostly involve pediatric patients. Likewise, a case report of gastric necrosis secondary to intestinal occlusion due to adhesions in a gynecological patient with a history of cystadenoma. Currently, multiple etiologies of acute gastric distension have been postulated, some of which, in addition to those mentioned above, include small bowel obstruction, postoperative gastric distension, gastric cancer, and Nissen-type fundoplication with an overly tight wrap [6-8].

Although this combination of complications cannot be prevented, timely and adequate diagnosis and management achieve more favorable results, with a decrease in mortality. The pathogenesis of acute gastric dilatation remains unclear. Cases of atony, such as those seen in eating disorders, were first described in 1859. In these patients, the stomach remains atonic, and muscle atrophy occurs during prolonged fasting. Later, during a binge episode, acute gastric dilation is precipitated. Due to the condition of atony, vomiting is not possible [3,4].

The other accepted theory is mechanical, as in the case of "superior mesenteric artery syndrome," in which acute gastric dilation is secondary to other diseases as follows: pancreatitis, peptic ulcer, appendicitis, and finally, vascular causes [9]. Gastric perforation and necrosis are the serious consequences of acute gastric dilation. The abundant blood supply that the stomach receives from the intramural anastomosis networks protects it against hypoperfusion, it is reported in cadavers that there is an adequate irrigation of the gastric wall only with a major permeable artery. However, increased intragastric pressure above 20 cm H_2_O has been reported to cause mucosal and submucosal ischemia. There is no consensus on the pressure threshold to cause gastric necrosis as studies have only been conducted in animals [5,13].

Another factor that is added is vascular insufficiency, in our patient, it could probably be conditioned by previous fundoplication surgery since it has been reported as a late complication and is generally triggered by some other condition of obstruction of transmural blood flow such as intestinal obstruction [14,15]. Gastric bubble syndrome presents as the inability of gas to pass from the stomach to the esophagus. Technical factors in fundoplication affecting "gas-bloat" syndrome, a cuff smaller than 2 cm, or systematic dissection of the short vessels of the stomach have been studied. However, there are no studies that define the role of resection of short vessels of the stomach and susceptibility to ischemia under hypoperfusion conditions. The sensitivity of the gastric body and fundus to ischemia has been demonstrated in experimental models, unlike the antrum which is less susceptible to ischemia, probably due to the supply of the gastroduodenal artery [16,17].

Some of the clinical manifestations of acute gastric distension are bleeding from the upper gastrointestinal tract, acute-onset abdominal pain, and nausea. Acute gastric dilatation alters the gastroesophageal junction and creates an angulation of the esophagus, making the possibility of emesis difficult, thus worsening the clinical picture [4-6].

The first diagnostic study to consider is the abdominal X-ray, in which acute gastric dilation may be evident, then an abdominal tomography can help us determine a cause or inform us about signs of gastric necrosis such as gastric pneumatosis, as presented by the patient reported in this report. Tomographic signs of perforation may be present, including intraperitoneal free air or obstruction with air-fluid levels in the intestine [6].

In most case reports, the location of gastric ischemia in the greater curvature and gastric fundus predominates, unlike the minor curvature and pylorus are usually respected [1-5,7-10]. Initial treatment always includes gastric decompression with a nasogastric tube, which releases a significant volume of fluid, including gastric contents or blood, often presenting as "coffee grounds." Fluid resuscitation is also initiated. In most of the reported cases, the definitive treatment is surgical, since a delay in the surgical intervention impacts mortality of up to 50-80% [5,18].

Some cases of acute gastric distention without necrosis resolve with nasogastric tube decompression alone; however, in some patients treated only with gastric decompression or placement of a gastrostomy tube, late gastric necrosis may develop. It has even been reported after up to 11 days of decompressive treatment and resolution of the underlying cause. Therefore, if conservative treatment is chosen, follow-up and monitoring with endoscopic diagnostic methods should be implemented, as they can be an effective option for ongoing evaluation [14,19,20]. 

In cases of gastric necrosis, the most effective treatment reported is partial or in some cases total gastrectomy. In most case reports, mechanical partial gastrectomy was performed with adequate postoperative evolution without early complications [12]. The existing evidence regarding acute gastric dilation and gastric necrosis is limited to case reports, due to the rarity of this condition. Therefore, we believe the importance of diagnosing it lies in the unique characteristics of the clinical presentation and its progression, as well as the outcomes of treatments provided in a timely manner.

Our patient presented a clinical picture of acute abdomen secondary to intestinal occlusion, evidenced in the corresponding clinical and imaging studies. With the aforementioned antecedents, the hypothesis in our patient is as follows: acute dilation and consequent gastric necrosis may have arisen from the inability to relieve intragastric pressure due to previous fundoplication, which resulted in a decrease in the vascularity of the gastric fundus, as well as the greater curvature of the stomach.

## Conclusions

The surgical treatment provided was appropriate, guided by clinical signs of peritoneal irritation, laboratory findings, and imaging results. Although the exact etiology remains unclear, we hypothesize that the patient's history of Nissen fundoplication, combined with distal intestinal obstruction, was a key factor contributing to the development of acute gastric dilation (AGD) and subsequent gastric ischemia. Partial gastrectomy, recognized as the treatment of choice for gastric necrosis based on the reported cases, was successfully performed in this patient, highlighting the importance of timely surgical intervention in similar cases.
